# Deficiency of CCN5/WISP-2-Driven Program in breast cancer Promotes Cancer Epithelial cells to mesenchymal stem cells and Breast Cancer growth

**DOI:** 10.1038/s41598-017-00916-z

**Published:** 2017-04-27

**Authors:** Amlan Das, Kakali Dhar, Gargi Maity, Sandipto Sarkar, Arnab Ghosh, Inamul Haque, Gopal Dhar, Snigdha Banerjee, Sushanta K. Banerjee

**Affiliations:** 1 Cancer Research Unit, Kansas City VA Medical Center, Kansas City, MO USA; 2 Department of Pathology & Laboratory Medicine, University of Kansas Medical Center, Kansas City, KS USA; 3 Division of Hematology and Oncology, Department of Medicine, University of Kansas Medical Center, Kansas City, KS USA; 4 Department of Anatomy and Cell Biology, University of Kansas Medical Center, Kansas City, Kansas USA; 5 Department of Biotechnology, Calcutta University, 35 Ballygunge Circular Road, Kolkata, India; 6 Syngene International Ltd, Clinical Development, Tower 1, Semicon Park, Phase II, Electronics City, Hosur Road, Bangalore, 560100 Karnataka India; 7 Biocon-Bristol Myers Squibb Research and Development Center (BBRC), Syngene International Ltd, Bangalore, 560099 Karnataka India

## Abstract

Breast cancer progression and relapse is conceivably due to tumor initiating cells (TICs)/cancer stem cells. EMT (epithelial-mesenchymal-transition)-signaling regulates TICs’ turnover. However, the mechanisms associated with this episode are unclear. We show that, in triple-negative-breast cancer (TNBC) cells enriched with TICs, CCN5 significantly blocks cellular growth via apoptosis, reversing EMT-signaling and impairing mammosphere formation, thereby blocking the tumor-forming ability and invasive capacity of these cells. To corroborate these findings, we isolated tumor-initiating side populations (SP) and non-side population (NSP or main population) from MCF-7 cell line, and evaluated the impact of CCN5 on these subpopulations. CCN5 was overexpressed in the NSP but downregulated in the SP. Characteristically, NSP cells are ER-α positive and epithelial type with little tumorigenic potency, while SP cells are very similar to triple-negative ones that do not express ER-α- and Her-2 and are highly tumorigenic in xenograft models. The overexpression of CCN5 in SP results in EMT reversion, ER-α upregulation and delays in tumor growth in xenograft models. We reasoned that CCN5 distinguishes SP and NSP and could reprogram SP to NSP transition, thereby delaying tumor growth in the xenograft model. Collectively, we reveal how CCN5-signaling underlies the driving force to prevent TNBC growth and progression.

## Introduction

Breast cancer (BC) remains one of the deadliest and most commonly identified malignant diseases in women in Western countries. It attacks one in eight women, impacting nearly every family worldwide. Despite extensive progress in diagnosis and treatment of BC, several clinical and scientific problems remain unresolved. As a result, treatments of advance stages of this disease are still fairly limited and ineffective^1^. The limitation of these therapy regimens is due to not yet effectively targeting two important events including epithelial to mesenchymal transition (EMT)^2–5^ and tumor initiating cells (TICs)/cancer stem cells (CSCs) turnover^5, 6^. These two features of cancer cells are interlinked with each other and play critical roles in BC progression and relapse^4, 6–9^. Based on pathology and gene expression profiling, triple negative (ER−, PR−, HER2−) breast cancer cells (TNBCs) are heterogeneous in nature and enriched with TICs/CSCs^1, 10^. These pathobiological settings make TNBC cells aggressive and less sensitive to standard chemotherapy. In recent years, the intra-tumor heterogeneity in BC has been shown to denote the co-habitation of sub-population of morphologically, genetically and interactively heterogeneous cancer cells. One of the sub-populations could be TNBC type and thereby intra-tumor heterogeneity poses a challenge for diagnosis and treatment^1, 11, 12^. Thus, a better understanding regarding the mechanisms that program EMT and stemness in these cells are likely critical in designing improved therapies of TNBC as well as heterogeneous tumors.

Like real-life tumors, heterogeneity in genetically clonal cell lines is a rule rather than exception^13^. MCF-7, an estrogen receptor positive BC cell line, is one of the best examples in BC research in which mixed bag of heterogeneous cell populations are well characterized. Two sub-populations, which are designated as main population (MP) or non-side population (NSP) and side population (SP), appear spontaneously in proliferating MCF-7 cells with various fractions^14–16^. The MP/NSP represents 97–99% of the populations and the remaining cells are SP cells. Identification and isolation of SP cells from the main population is based on the increased capacity of the sub-population of cells to efflux out the Hoechst dye and similar lipophilic dyes via ATP-binding cassette (ABC) transporter proteins which are localized in their cell membrane^17^. The SP cells of both human and murine origin showed higher efficiency of dye efflux compared to the remaining NSP cells, and proven to be enriched with TICs/CSCs^18–22^. Global characterization of transcriptosomes in SP and NSP/MP cells found distinct expression levels of different genes in these subpopulations of cancer cells demonstrating that SP cells are less differentiated than NSP/MPs and display similarities to TNBC/TICs cells^23^ and may suggest that they originate from same the precursor cells in the differentiation process. However, the mechanism of propagation SP cells from NSP/MP or precursor cells has not yet been fully revealed.

CCN5 (also known as Wnt-1-induced signaling protein-2 or WISP-2) is a 24–31-kDa matricellular protein that acts as a negative regulator of BC progression^24^. CCN5 is found to be constitutively expressed in less aggressive human BC cells (i.e. MCF-7 and ZR-75-1), whereas its expression is minimally detected in moderately aggressive BC cell lines (i.e. SKBR-3) and it is completely undetected in the highly aggressive BC cell line (i.e. MDA-MB-231)^21, 24^. CCN5-signaling has been found to prevent invasiveness and progression of the disease^24–28^, and the anti-invasive role of CCN5 has been shown to be mediated by inhibition of miR10b through HIF-1α-TWIST signaling via regulation of EMT^29^. Moreover, our and other group studies implicate that CCN5 depletion by introducing genetic lesions such as mutational activation of mutant p53, TGF-β activation or by RNAi-based approaches makes ER+ BC cells more aggressive^25, 30^. One of the previous studies, however, found that CCN5 depletion suppressed SP turnover in MCF-7 cells but enhanced tumor growth progression in the MCF-7-xenograft^25^, suggesting that an atypical consensus on selection criteria of reprogramming of CCN5 action exists. Thus, it is needed to differentiate whether the cells deficient of CCN5-signaling assumes TIC-like behavior and forms aggressive breast tumors in xenograft model.

To address the unmet issues and establish the anti-invasive role of CCN5, in this study, we selected TNBC cell lines (i.e., MDA-MB-231 and HCC-70), which are an enriched source of TICs with stem cell properties and isolated SP and NSP cells from MCF-7, and we examined the impact of CCN5-signaling on functions and survival of these cells under *in vitro* and *in vivo* settings using human recombinant CCN5 protein (hrCCN5) treatment and genetic manipulation. These studies found that CCN5 suppressed tumor growth by apoptotic signaling pathway. In addition, CCN5 restored ER-α, reprogramed mesenchymal-epithelial transition (MET) and blocks TIC’ turnover that may impair tumor progression.

## Results

### CCN5 protein treatment selectively inhibits cell proliferation via apoptosis in TNBC Cells

**Figure 1:**
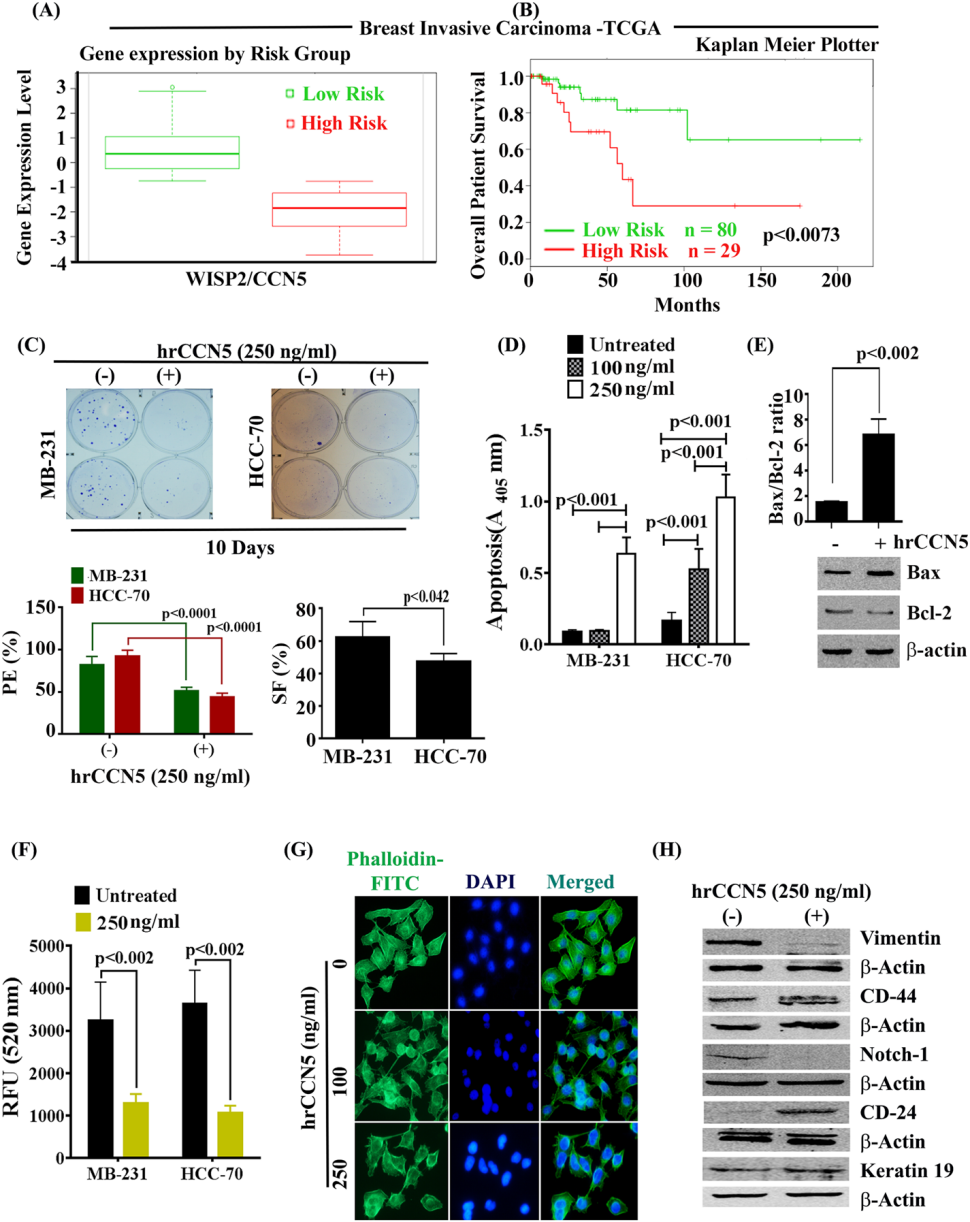
CCN5 overexpression is associated with good prognosis of human breast cancer and CCN5-treatment suppresses growth and invasive phenotypes of TNBC cells. (**A**) Plots generated by SurvExpress program to analyze samples of breast invasive carcinoma patients from TCGA show the expression levels of CCN5. Low- and high-risks groups are shown in green and red, respectively. (**B**) Kaplan-Meier overall survival curves of CCN5 signatures were constructed by using the SurvExpress program and the P-value resulting from a t-test of the difference. Low- and high-risks groups are shown in green and red, respectively. (**C**) Effect of hrCCN5 or vehicle alone (control) on cell survival of TNBC cells (i.e., MDA-MB-231 and HCC-70) was measured after 48 h using anchorage-dependent growth (ADG)/Colony/focus forming assay. We determined the plating efficiency (PE) (left panel) and survival fraction (SF) (right panel) in both cell lines as described in Materials and Methods. Upper panel is the representative images of ADG in different treatment setups. Bar graphs indicate PE (left) and SF (right) in hrCCN5-treated or control samples. Error bars indicate mean ± SD, and represent at least three independent experiments. (**D**) Dose-dependent effect of hrCCN5 on apoptotic cell death was measured in TNBC cells after 48 h using cell-death detection ELISA kits. Error bars indicate mean ± SD, and represent at least three independent experiments. (**E**) Representative Western blots and quantification (bargraph) of Bax/BCl-2 ratio in the lysates of hrCCN5-treated or vehicle-treated MDA-MB-231 cells. β-actin was used as loading controls. Error bars indicate mean ± SD, and represent at least three independent experiments. (**F**) Effect of hrCCN5 or vehicle alone on aggressive malignant phenotypes of TNBC cells was measured by anchorage-independent growth (AIG) using fluorescence-based soft agar colony forming assay. Error bars indicate mean ± SD, and represent at least three independent experiments. (**G**) Detection of morphological alteration from mesenchymal to epithelial type in MDA-MB-231 cells following hrCCN5 treatment using Phalloidin-FITC. (**H**) Representative Western blots of EMT markers in the lysates of hrCCN5-treated or vehicle-treated MDA-MB-231 cells. β-actin was used as loading controls. Statistical significance was determined using long rank test, two-way ANOVA and two-tailed unpaired Student’s t-test. All the photographs are cropped from original figures.

The previous studies have suggested an anti-invasive role of CCN5 in aggressive TNBC cells^24, 29, 31, 32^. While examining the relationship of CCN5 mRNA expression levels and clinical outcome of human BC progression using bioinformatically analyzed (SurvExpress program) multiple published global microarray datasets of BC patients with or without receptors (i.e., ER, PR and HER-2)^33^, we found high expression of CCN5 predicts good prognosis and increase overall patient survival (Fig. 1A and B). Collectively, these results further strengthening the previous concept that CCN5 is a negative regulator of BC progression^24, 26, 31^.

Our next goal was to determine the impact of CCN5 on TNBC biology. We first probed the efficacy of hrCCN5, which has been validated before use by Western blotting showing a single immuno-reacted ~24 kD band, Figure S1), on *in vitro* cellular proliferation of TNBC cells such as MDA-MB-231 and HCC-70. In addition, hrCCN5 effect was also tested in CCN5-positive MCF-7 cells to provide evidence that hrCCN5 has no effect on the cells that endogenously express CCN5. Consistent with previous work^32^, a significant reduction in proliferation of MDA-MB-231 cells and HCC-70 (Figure S2) were observed in dose dependent manner. Half maximal inhibitory concentration (IC_50_s) of hrCCN5 in both cell lines was 250 ng/ml or high. No detectable effect was observed in MCF-7 cells (Figure S2). The colony formation assay/*in vitro* cell survival assay shows morphologically distinct colonies formed by MDA-MB-231 and HCC-70 cells but the effect of CCN5 on colony forming ability of these cells were identical. CCN5-treatment significantly reduced the plating efficiency (PE) and survival fraction (SF) in both cell lines (Fig. 1C). Growth inhibition of TNBC cells by hrCCN5 is mediated through the induction of apoptosis in a dose-dependent fashion (Fig. 1D), and it could be mediated through the regulation of Bcl-2/Bax pathway as Bax/Bcl-2 ratio was significantly increased in hrCCN5 treated MDA-MB-231 cells compared to untreated cells (Fig. 1E). While both TNBC cells are sensitive to hrCCN5, response of HCC-70 to hrCCN5 was significantly higher than MDA-MB-231 cells.

### CCN5 controls *in vitro* Transformation and Aggressiveness of TNBC cells

The anchorage-independent cell growth (AIG) has been considered a unique property of cancer cells as this phenomenon is linked to, as well as a surrogate marker for cancer cell aggressiveness such as tumorigenic, invasion and metastatic potentials^34^. To define the role of CCN5 in controlling transformation and aggressiveness of TNBC cells, AIG assay was performed. The studies showed that AIG was significantly reduced in hrCCN5 treated MDA-MB-231 and HCC-70 cells (Fig. 1F).

Phenotypical alteration such as epithelial to mesenchymal transition (EMT) is a hallmark of the aggressive progression of cancer and involves acquisition of stem cell-like features^35^. To assess the effect of CCN5 on EMT, MDA-MB-231 cells were treated with different doses of hrCCN5 protein and then morphology as well as molecular markers was studied. We have shown that hrCCN5 treatment significantly reversed the EMT and decreased the expression of mesenchymal markers (such as Vimentin, Notch1) and stem cell markers (such as CD44) while the epithelial markers (such as Keratin-19 and CD24) are found to be significantly up regulated after hrCCN5 exposure (Fig. 1G and H).

**Figure 2:**
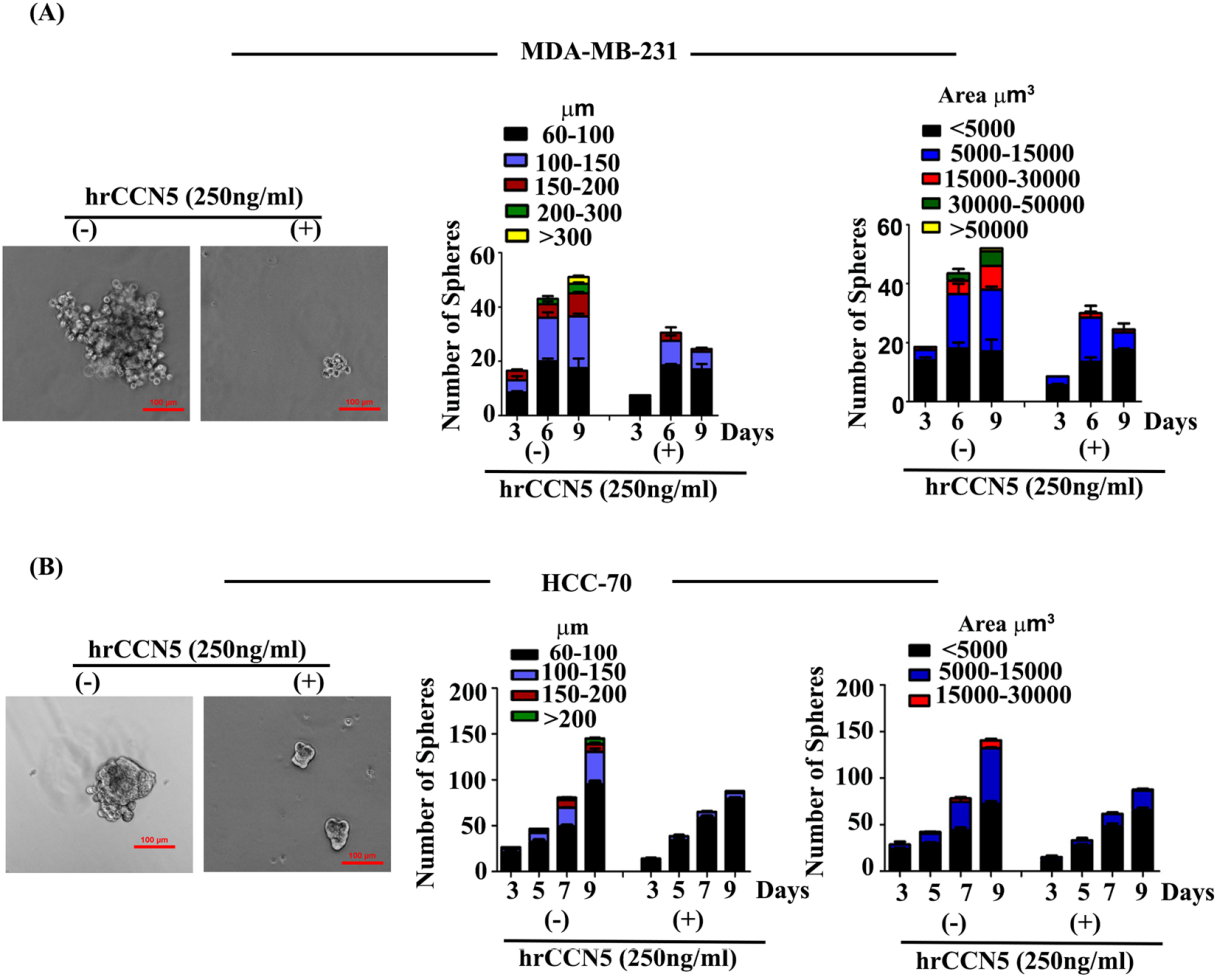
CCN5-treatment suppresses sphere-forming ability and self-renewal of tumor initiating cell (TICs) population of TNBC cells. (**A**,**B**) Effect of hrCCN5 or vehicle alone (control) on the mammosphere forming ability of MDA-MB-231 and HCC-70 was determined at different time points indicated in the Figure. Photographs illustrate structure and size of spheres in hrCCN5-treated and control cells. Bar graph represents the distribution of mammospheres of different sizes (left) and area (right) in treated and untreated groups. Scale bar = 100 μm. Error bars indicate mean ± SD, and represent at least three independent experiments. Statistical significance was determined using two-tailed unpaired Student’s t-test.

For further corroboration of the antitumorigenic role of CCN5 in TNBC, we determined the effect of CCN5 treatment in CSCs/TICs status, which is responsible for tumor initiation, development and also recurrence of the disease. To do so, we performed *in vitro* mammosphere assays. Upon treatment of hrCCN5 for 7days, MDA-MB-231 and HCC-70 display a significant reduction in sphere number with different sizes (such as 60 μm to 300 μm or above) and areas compared to untreated controls (Fig. 2). Moreover, the effect of CCN5 was markedly greater in HCC-70 relative to MDA-MB-231.

**Figure 3:**
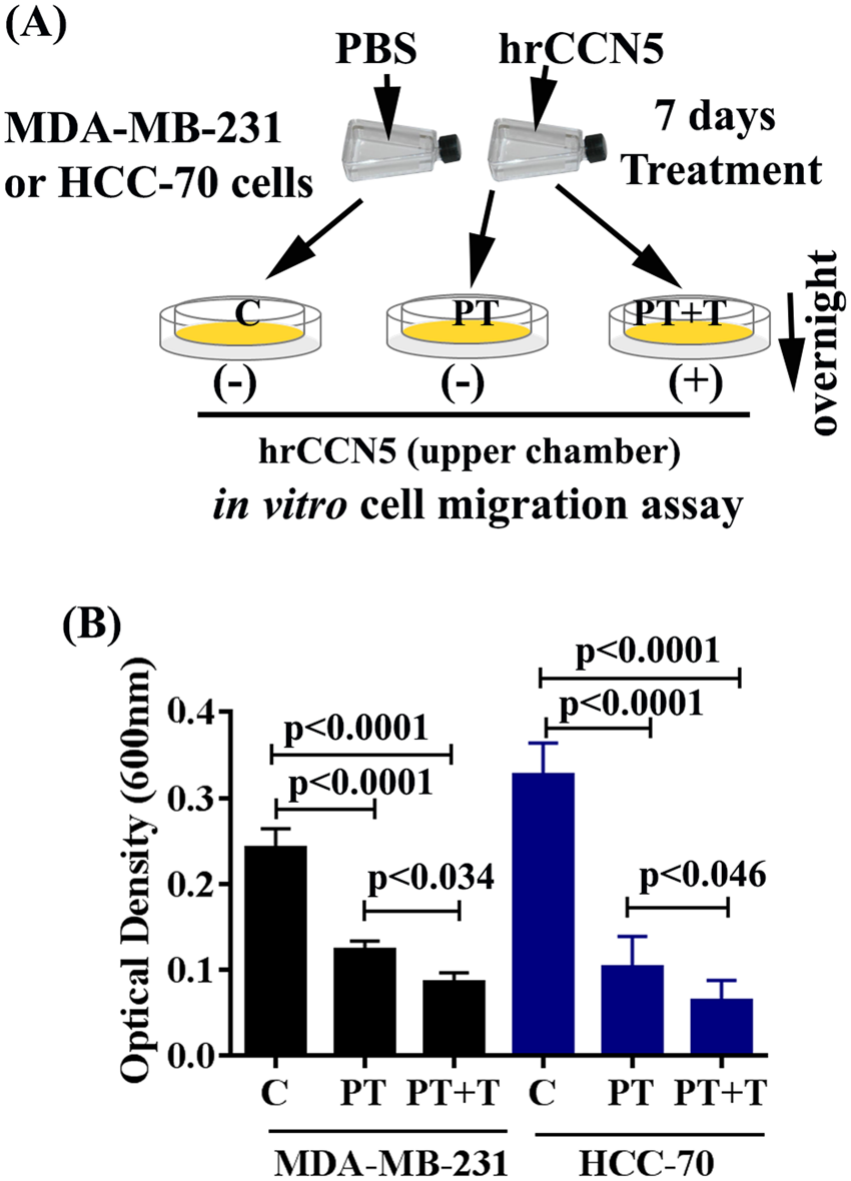
CCN5 treatment prevents *in vitro* migration of TNBC cells. (**A**) The schematic representation of experimental design. Cells were treated with hrCCN5 (250 ng/ml) or vehicle for 7 days and then cells (10,000 cell/well) were seeded on the upper chamber of the modified Boyden chamber in the presence (PT + T) or absence (C or PT) of hrCCN5 for overnight migration towards serum. C, control, T, treatment and PT, pre-treated. Individual figures (i.e., T-flask and petri-disk) were obtained from ScientificSlides suite, a Microsoft PowerPoint based software. (**B**) The bar graph represents the quantification of migration efficiency in hrCCN5-treated and untreated groups. The extent of migration was measured according to the protocol indicated in the Materials and Methods section. Data show mean ± SD, and are representative of at least three independent experiments. Statistical significance was determined using two-tailed unpaired Student’s t-test.

Since EMT and cancer stemness are associated with invasion and solid tumor progress^36–39^, we measured the effect of CCN5 treatment on TNBC cell *in vitro* migration using modified Boyden chamber assay. The results showed that like SP cells, migration of hrCCN5-pretreated MDA-MB-231 and HCC-70 cells were significantly less relative to controls (Fig. 3), and correlates with the EMT and stemness reversal by CCN5 treatment.

### CCN5 is Deficient in MCF-7 Side Population and Upregulation of CCN5 Promotes Mesenchymal to Epithelial Transition (MET) and Impairs Stemness

**Figure 4:**
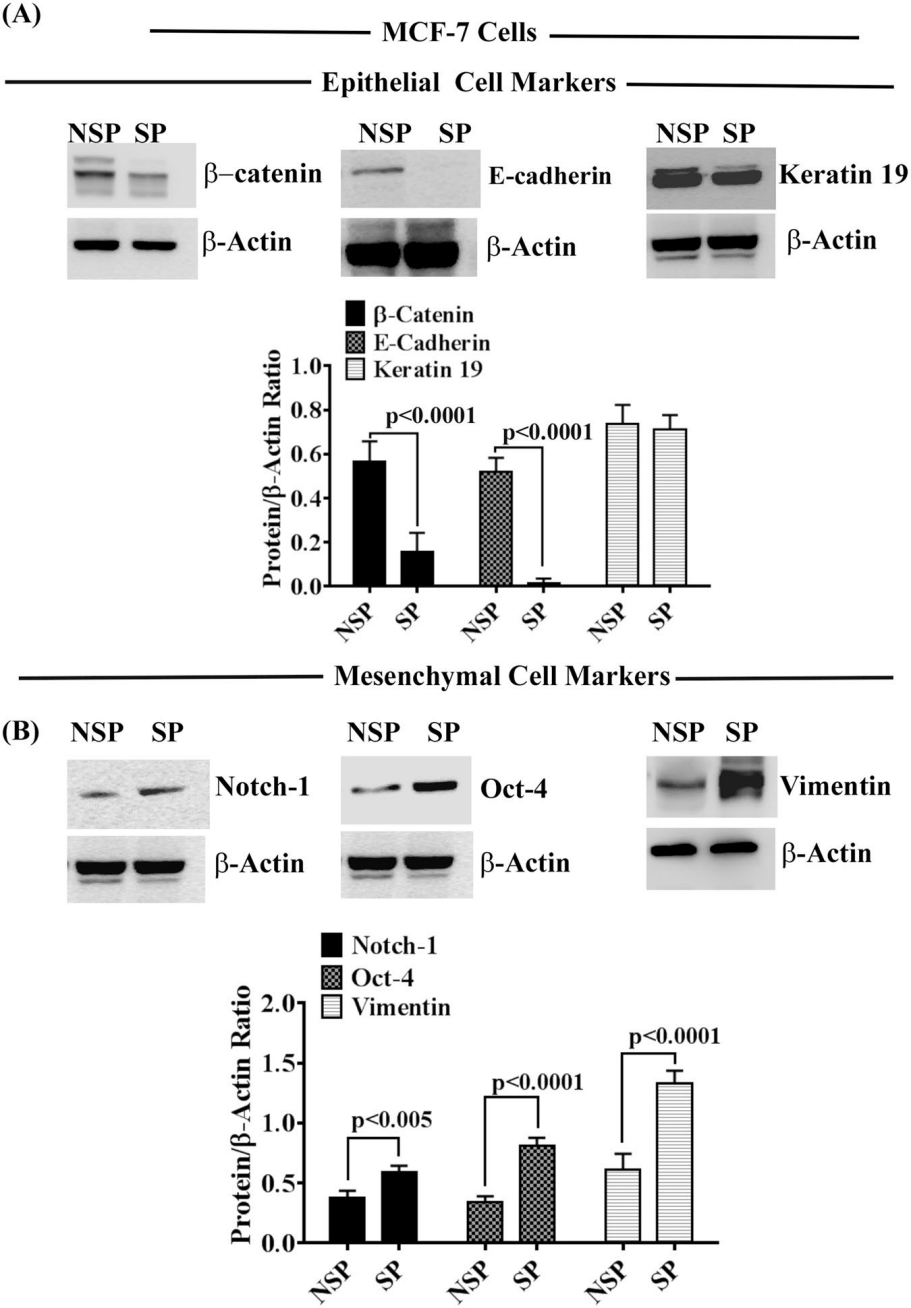
Differential expression of epithelial and mesenchymal markers in side population (SP) and non-side population (NSP) in MCF-7 cell line. (**A**,**B**) Western blot analysis and quantification of epithelial and mesenchymal/stem cell markers in SP and NSP cells isolated from MCF-7 cell line. Data show mean ± SD, and are representative of at least three independent experiments. Statistical significance was determined using two-tailed unpaired Student’s t-test. All the photographs are cropped from original figures.

**Figure 5:**
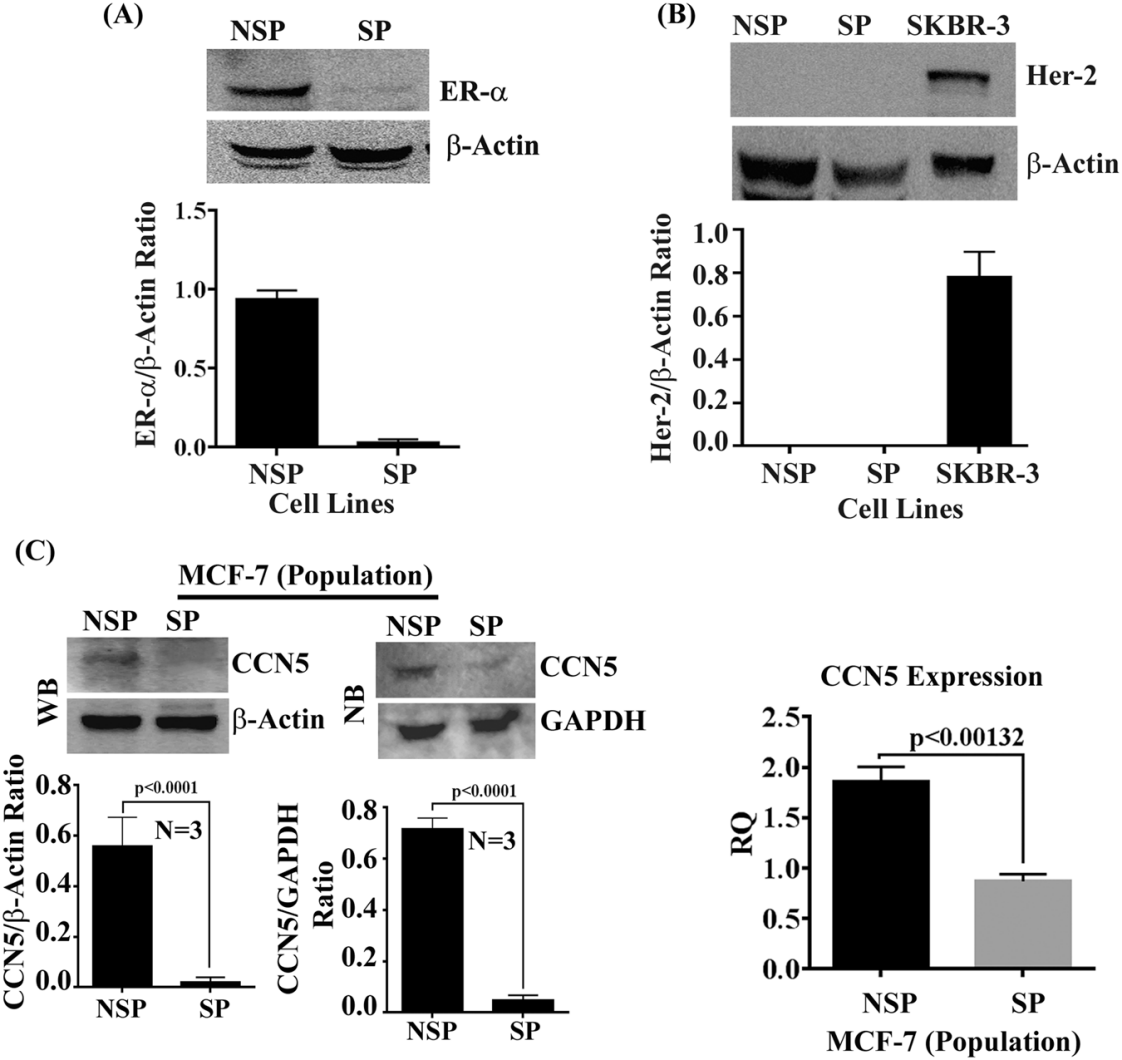
Expression profiles of ER-α, Her-2 and CCN5 in MCF-7 heterogeneous cell populations. (**A**) ER-α Status. Detection and quantification (bargraph) of ER-α protein level in NSP and SP cells isolated from MCF-7 cells using Western blot analysis. β-actin is used as a loading control. (**B**) Her-2 status. Detection and quantification (bargraph) of ER-α protein level in NSP and SP cells isolated from MCF-7 cells using Western blot analysis. β-actin is used as a loading control. (**C**) CCN5 status. Western blot analysis (upper and middle left panels), Northern Blot analysis (Upper and middle right panels) and qRT-PCR (lower panel) for the quantification of CCN5 expression in NSP and SP cells isolated from MCF-7 cell line. Data show mean ± SD, and are representative of at least three independent experiments. Statistical significance was determined using two-tailed unpaired Student’s t-test. All the photographs are cropped from original figures.

**Figure 6:**
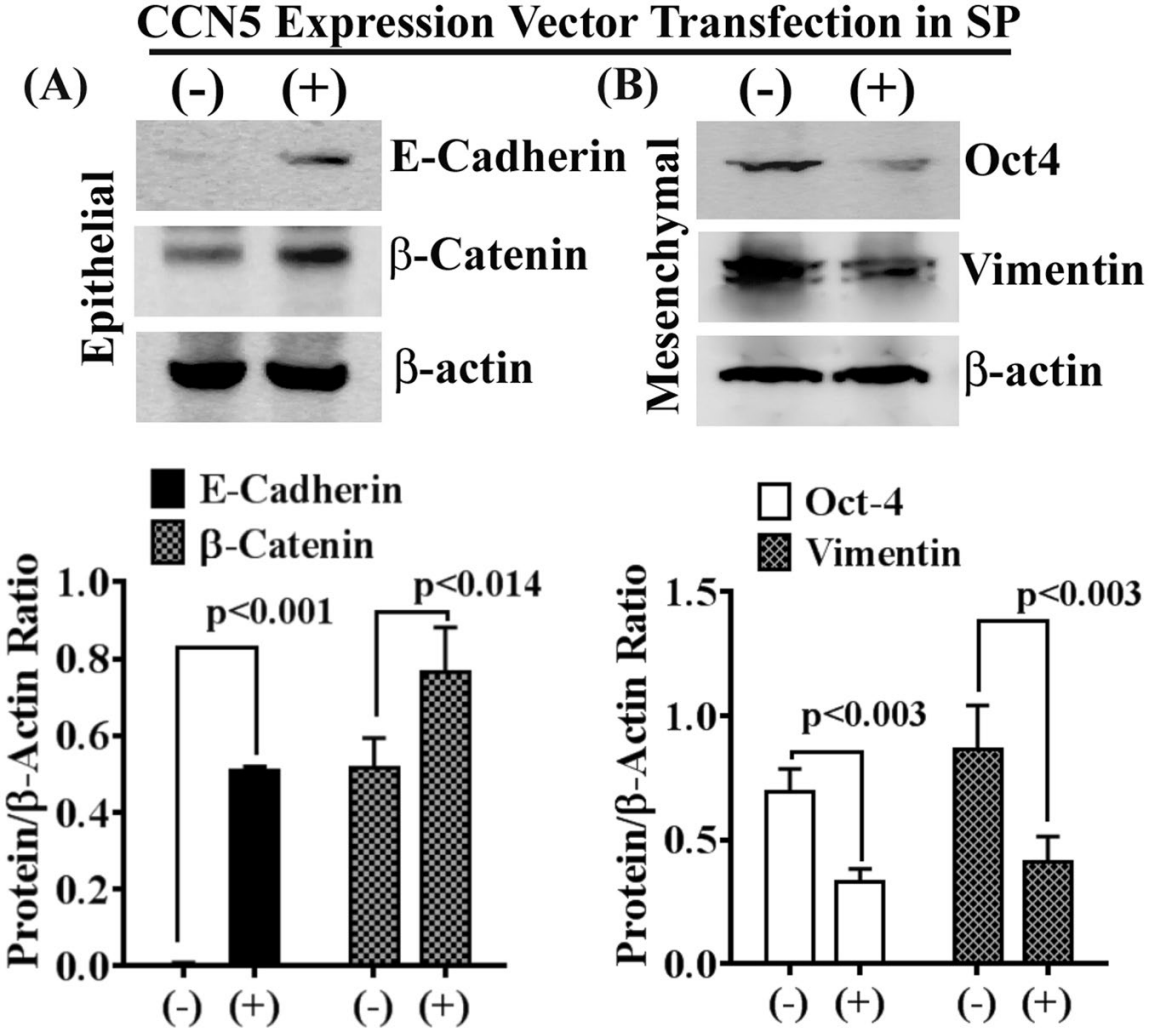
CCN5 overexpression suppresses EMT program in SP of MCF-7 cells. (**A**,**B**) Western blot analysis and quantification of epithelial (**A**) and mesenchymal (**B**) markers in SP cells stably transfected with vectors containing CCN5 gene or vector alone. Data show mean ± SD, and are representative of at least three independent experiments. Statistical significance was determined using two-tailed unpaired Student’s t-test. All the photographs are cropped from original figures.

Using MCF-7-SP cells as a model for TICs/CSCs, we investigated the status of CCN5 in SP and NSP descendants (constituent cancer cells). To do so, first, we isolated SP and NSP cells from MCF-7 cells according to our protocol^22^, and cultured them in appropriate culture conditions (Figure S3). The SP and NSP cells were characterized by determining the levels of different molecular markers for epithelial, mesenchymal and stemness in the cell lysates using Western blotting (Figs 4 and S4). Consistent with previous work^25^, we found that the isolated SP cells weakly expressed epithelial markers such as β-catenin and E-cadherin, while mesenchymal/stem cell markers are strongly expressed in SP as compared to NSP. The studies find that the surface markers CD24 and CD44 proteins are differentially expressed in SP cells (Figure S4). In SP cells, CD44 is highly expressed while CD24 minimally expressed as compared to NSP cells. Moreover, we found that the SP cells are estrogen receptor-alpha (ER-α) and Her-2 negative (Fig. 5A and B). After characterization of SP and NSP cells, we next examine the status of CCN5 mRNA and protein levels using qPCR, Northern blotting and Western blotting. CCN5 mRNA/protein expression was found significantly less in MCF-7-SP cells compared to Non-SP cells (Fig. 5C and D). Next, we aimed to experimentally characterize the specific contribution of CCN5 deficiency in maintaining the mesenchymal/stem cell features of SP cells. We engineered SP cells expressing CCN5 gene (SP^CCN5T^) or expression vector alone (control). Compared with controls, constitutive ectopic expression of CCN5 (SP^CCN5T^) caused significant reversion in expression of EMT markers (Fig. 6). In contrast, suppression of CCN5 expression by small hairpin RNA (shRNA) in NSP cells results EMT progression in these cells (Figure S5). Collectively, these studies indicate that CCN5 controls SP and NSP in MCF-7 cells and in absence of CCN5, SP cells behave like TNBC.

### CCN5 regulates ER-α expression and activity in subpopulation of MCF-7 cells

**Figure 7:**
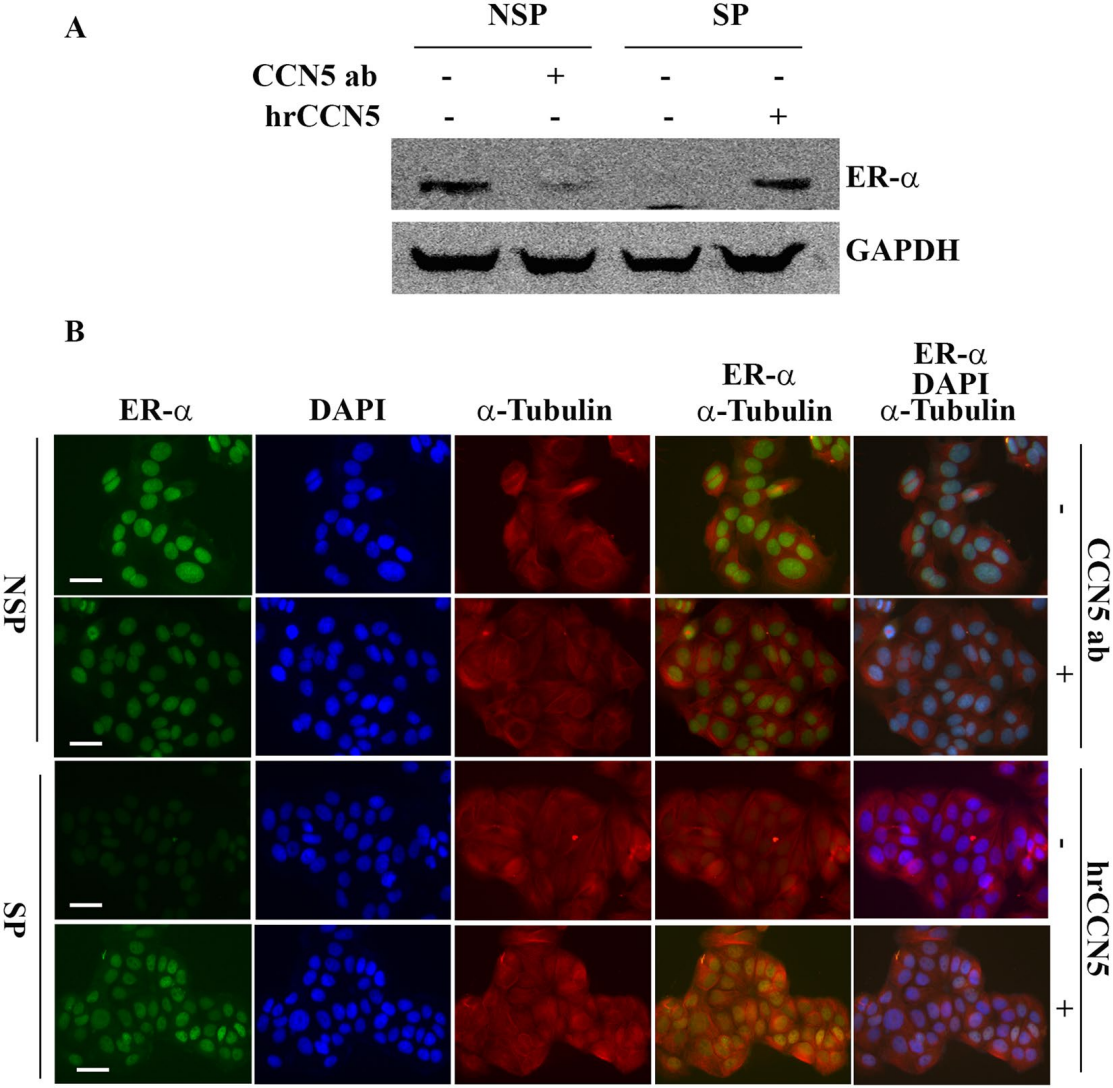
CCN5 regulation of ER-α in subpopulation of MCF-7 cells. (**A**) The Western blot analysis represents the expression of pER-α in NSP cells treated with anti-CCN5 antibody treatment (500 ng/ml) or PBS and SP cells treated with hrCCN5 protein (250 ng/ml) treatment or PBS for 72 h. GAPDH was used as a loading control. (**B**) Florescence microscopy for ER-α in NSP cells with or without CCN5 antibody (500 ng/ml) treatment and SP cells with or without hrCCN5 protein (250 ng/ml) treatment for 72 hours. ER-α was stained by indirect immunofluorescence, using a FITC-conjugated secondary antibody. α-Tubulin was stained using Alexa Fluor 555-conjugated antibody. First, second and third columns show staining for ER-α, DAPI and α-Tubulin respectively. Merge images of ER-α and α-Tubulin (fourth column) document lower expression of ER-α upon anti-CCN5 antibody treatment in MCF7-NSP cells and elevated ER-α expression after hrCCN5 protein treatment in MCF7-SP cells. Scale bar = 50 μm.

Because CCN5 expression is detected in ER-α positive NSP cells, it is of interest to investigate whether CCN5 is involved in regulation of ER-α expression in these sub-populations. To this end, we treated SP cells with hrCCN5 and NSP cells were exposed to CCN5 antibody to block CCN5 action. We found that phospho-ER-α (pER-α) expression was induced in SP cells by hrCCN5 treatment while pER-α expression was impaired in NSP cells following CCN5 antibody treatment (Fig. 7). These results were confirmed by Western blot (Fig. 7A) and immunofluorescence (Fig. 7B) analyses. Collectively, this study indicates that CCN5 is a regulator of ER-α in BC cells.

### CCN5 controls SP cell Proliferation, migration, and Tumor Progression

**Figure 8:**
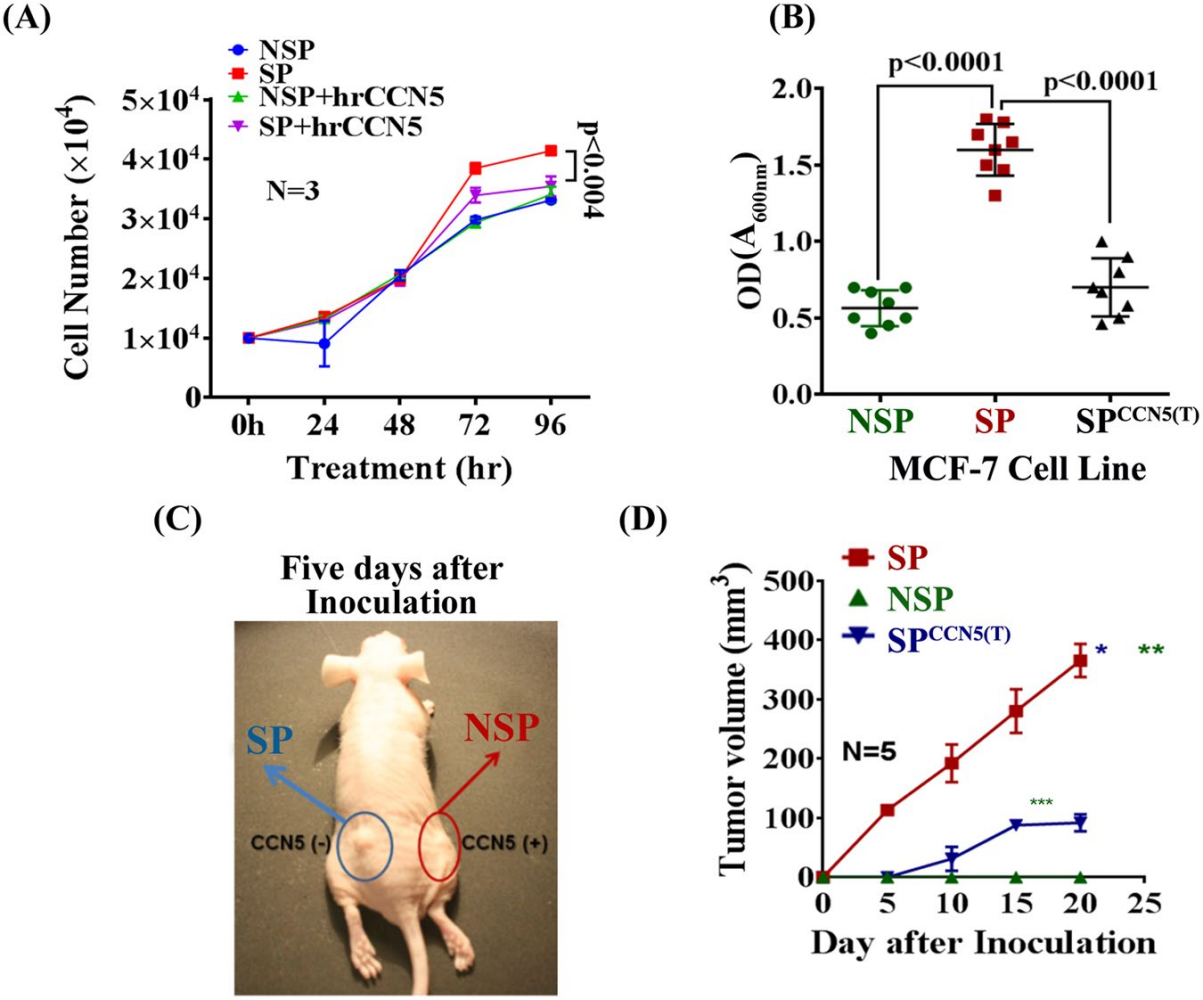
CCN5 prevents *in vitro* SP cell proliferation, migration and tumor regression *in vivo*. (**A**) Cell growth analysis. Cell growth analysis of NSP and SP in the presence or absence of hrCCN5 (250 ng/ml) was measured in different time points as indicated in the Figure by cell counting. Error bars indicate mean ± SD, and represent at least three independent experiments. (**B**) *In vitro* migration assay. NSP, SP and CCN5-transfeted SP (SP^CCN5(T)^) cells were seeded on the upper chamber of the Boyden chamber and allowed them to migrate towards serum overnight. The extent of migration was measured according to the protocol indicated in the Materials and Methods section. Data show mean ± SD and are representative of at least eight independent experiments. (**C**) Representative subcutaneous tumor xenograft nude mouse model exhibiting tumor growth by SP cells within 3–5 days following inoculation while no sign of tumor growth in the NSP cell-inoculation site. (**D**) Tumor growth curves of NSP, SP and SP^CCN5(T)^ cells. Growth curve was plotted by measuring the relative tumor volume in different time points as indicated in the graph. Error bars represent means ± SD (n = 5 mice per group). *p < 0.001(SP vs SP^CCN5(T)^), **p < 0.0001 (SP vs NSP), ***p < 0.0001 (SP^CCN5(T)^ vs NSP). Statistical significance was determined using two-tailed unpaired Student’s t-test.

Based on aforementioned data, we aimed to determine the contribution of CCN5 in proliferation, migration and tumor forming ability of SP cells and progression of those tumors. We found that SP cells were sensitive to CCN5. The cell proliferation was significantly decreased after hrCCN5 (250 ng/ml) treatment for 72 h compared to vehicle-treated cells (Fig. 8A). The *in vitro* migration of SP^CCN5(T)^ cells significantly less compared to SP^CCN5(−)^ (Fig. 8B). Interesting, it has been noticed that the NSP cells are insensitive to CCN5.

Based on these encouraging *in vitro* observations, we were intrigued to know the effect of CCN5 on SP cell-mediated tumor growth. The nude mice (N = 5) inoculated bilaterally with the SP and NSP or SP^CCN5(T)^ cells in left and right side of the body, palpable tumors developed within 3–5 days after injection and 80% of mice displaying frank tumor growth by 20 days, whereas NSP cells or SP^CCN5(T)^ cells showed no tumor development at the inoculation sites within 3–5 days (Fig. 8C and D). After 20 days of inoculation, while no tumor growth was identified in the inoculation sites of NSP cells, palpable tumors were appeared in the inoculation sites of SP^CCN5(T)^ cells but caused significant tumor growth retardation as compared to SP-tumor growth (Fig. 8D).

## Discussion

The basal like-TNBC is highly aggressive cancer with worst prognosis and has lack of effective targeted therapy^40^. In BC tumor heterogeneity, TNBC cells are frequently coexisted with mixed bag of cancer sub-populations including luminal-type (ER and PR positive)^1, 12, 41–43^. Thus, intra-tumor heterogeneity frequently poses a huge challenge for diagnosis and treatment selections^11, 12^. Understanding the underlying mechanism controlling the aggressive phenotypes and growth of TNBC cells are therefore critical and may uncover therapeutic interventions.

Having noted an adverse association between CCN5 and BC progression^24^ (Fig. 1A and B), we used MDA-MB-231and HCC-70 cell lines as *in vitro* TNBC model and side-population (SP)-model of MCF-7 cells as a mean to interrogate whether deficiency of CCN5-driven program in BC promotes cancer epithelial cells to mesenchymal stem cells and BC growth. In TNBC model, we found comprehensive pattern of CCN5 effect than that previously reported^24, 31, 32^. CCN5-treatment significantly reduces the TNBC cell growth parallel with induction of apoptosis and Bcl-2/Bax ratio (Fig. 1), indicating that CCN5 suppresses the growth of TNBC cells via apoptosis possibly through the mitochondrial Bcl-2-Bax pathways. An essential inhibitory role of CCN5 in tumorigenic potential of TNBC cells as well as reversing the EMT program, stemness and anti-invasive behavior were found, demonstrating tumor suppressor role of CCN5 and simultaneously confirming earlier findings that CCN5 is an anti-invasive molecules^24^. However, as CCN5 has recently been shown to suppress metastatic growth in gastric cancer^28^, further studies, perhaps using appropriate animal models to explore the anti-metastatic role, are warranted.

A required role for CCN5 in inhibition of BC progression is similarly found in SP-MCF-7 model (Figs 4–6) to extend the antitumorigenic role in aggressive BC^24, 25, 32, 44, 45^. We observed that like TNBC cells, ER-α, Her-2 and CCN5 is down regulated in SP cells while MCF-7 as well as NSP of MCF-7 cells are ER-α and CCN5 positive. Further, we find that ectopic overexpression CCN5 in SP cells reverses EMT and stemness as documented by upregulations of epithelial controlling proteins such as E-cadherin, β-catenin and CD24 concurrently with downregulations of regulatory factors of mesenchymal and stemness such as Oct4, Vimentin and CD44. We also observed that CCN5 treatment or overexpression in SP cells significantly impairs *in vitro* growth, migration and tumor forming ability in xenograft model. Aside from inhibiting role of CCN5, we found that CCN5 restored ER-α expression and activity in SP cells as pER-α (Ser118), which is an active form of ER-α associated with cell cycle regulation^46^, is upregulated in SP cells. Collectively, these studies suggest that CCN5 blocks self-renewal capacity of SP cells as well as de-differentiates SP to constituent cancer cells (like NSP) via reversing EMT, stemness and restoring ER-α and thereby SP cells lost their tumor initiating skills.

**Figure 9:**
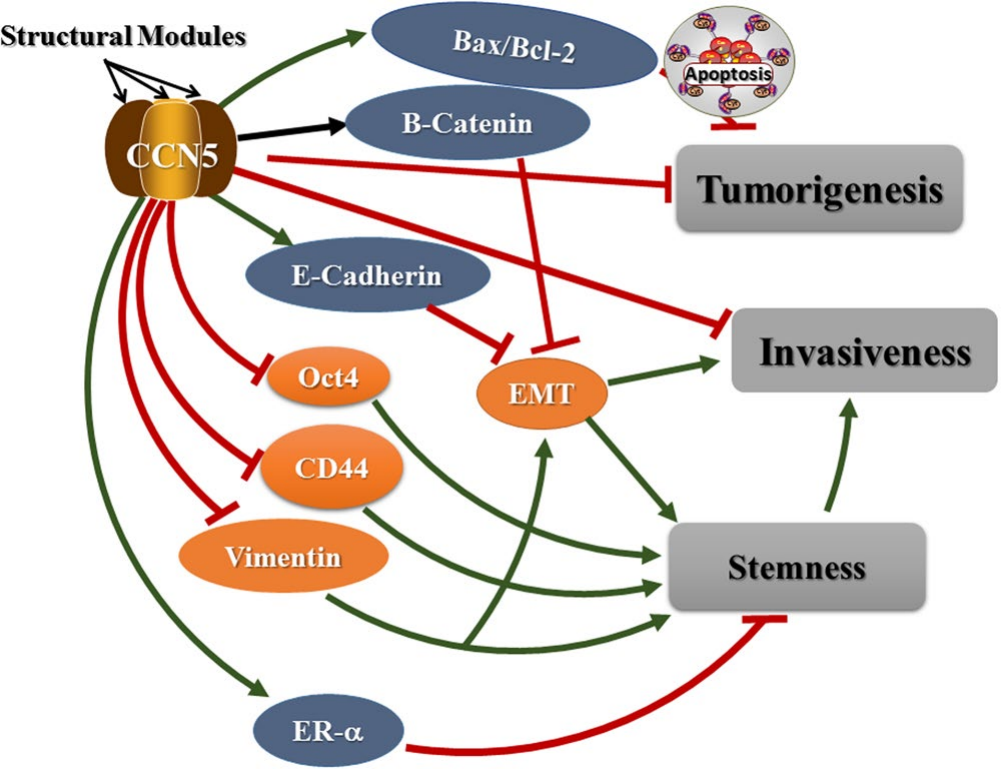
Diagram illustrating CCN5 regulation of molecular markers associated with cell growth, EMT and stemness in TNBC cells. Blue indicates upregulated proteins and orange indicates downregulated proteins. Individual figures were obtained from ScientificSlides suite, a Microsoft PowerPoint based software.

In summary, our studies illustrate how a single signaling pathway (CCN5-signaling) can control breast tumor growth and progression via regulating sequential, multistep molecular signatures that are linked with apoptosis, EMT program and stemness (Fig. 9). Thus, it is enticing to speculate that restoring of CCN5 or CCN5 protein treatment may exert therapeutic benefit in BC and possibly other cancers via regulating BC cell plasticity^28, 47–49^.

## Materials and Methods

### Cell Culture and Reagents

All experiments were performed using NSP and SP of MCF-7 cell line, MDA-MB-231 and HCC-70 BC cell lines. MCF-7, MDA-MB-231 and HCC-70 were purchased from ATCC (Manassas, USA). NSP and SP cells from MCF-7 cells were isolated and characterized in our core facilities. Details of culture conditions and procedures of these cell lines were described previously^26^. Unless stated otherwise, all reagents and chemicals were purchased from Sigma-Aldrich (St. Louse, MO, USA). Human recombinant CCN5 protein (hrCCN5) was obtained from PeproTech (Rocky Hill, NJ, USA) and purity was tested after every purchased using Western blot analysis (see Figure S1). CCN5 antibody was generated in our laboratory^50^. Fetal bovine serum (FBS) was obtained from ATCC (Manassas, VA, USA). Soft agar assay kit was purchased from Cell Biolabs, Inc. (San Diego, CA, USA). CD44 monoclonal antibody was purchased from Cell Signaling Technology, Inc. (Danvers, MA, USA), CD24 from Santa Cruz Biotechnology (Dallas, TX, USA), E-cadherin from BD Biosciences (Franklin Lakes, NJ, USA); Vimentin from Thermo Fisher Scientific (Waltham, MA, USA). The dilution of the antibodies was used as per manufacturer’s recommendation.

### Isolation of side population (SP) cells

The SP cells from MCF-7 cell line were isolated according to our previous methods^22^. Briefly, single-celled suspension of MCF-7 cells were resuspended in DMEM containing 10% FBS at concentration of 1 × 10^6^ cells/100 μl. Vybrant-Violet solution (10 μM) and ABCG inhibitor, Verapamil (50 μM) solution were added into the sample and incubated at 37 °C for 90 min. After incubation, cells were centrifuged, and resuspended in ice-cold 2 μg/ml propidium iodide in 1 x PBS to exclude dead cells before flow cytometry analysis. MCF-7 cells were analyzed by BD FACS Aria SORP flow cytometer (BD Biosciences) using ~405 nm excitation and 440 nm emission. After the SP and NSP cells were successfully separated, we re-analyzed the cells to evaluate sorting purity.

### Cell Number, Proliferation and Apoptosis

Cell number was measured using trypan blue exclusion on a cell counter device Cellometer Auto T4 Bright Field Cell Counter (Nexcelom Bioscience LLC, Lawrence, MA, USA). Cell viability/proliferation was measured by counting the cell numbers in using Crystal Violet staining followed by the measurement of absorbance at 600 nm using SOFTmaxPRO. Apoptosis was assayed using cell-death detection ELISA kits (Roche Diagnostic Corporation, Indianapolis, IN, USA).

### Transfection, Recombinant Protein Treatment and Western Blotting

Expression vectors containing CCN5 or expression vectors alone were obtained from OriGene Technologies, Inc. (Rockville, MD, USA) and transfected into SP cells using Lipofectamine 2000, according to manufacturer’s instructions. In addition, MDA-MB-231 and HCC-70 cells were treated with human recombinant CCN5 (hrCCN5) proteins with different doses (i.e., 100 ng/ml or 250 ng/ml) or vehicle alone. Cell lysis preparation from different experimental samples and Western blotting were performed as described^51^.

### qRT-PCR, Probe Preparation and Northern Blotting

Total RNAs (5 μg) from cultured cells were extracted using TRIzol reagent (Thermo Fisher Scientific, Waltham, MA, USA), and reverse transcribed with SuperScript II cDNA Synthesis kit Thermo Fisher Scientific (Waltham, MA, USA) according to the manufacturer’s instructions. qPCR was performed using the SYBR Green PCR master mix (Applied Biosystems, USA) and relative expression of the CCN5 mRNA was normalized to GAPDH. The cDNA probe was prepared followed by Northern blotting was performed according to the previous method^52^. The PCR primers for amplifying specific genes are previously described^52^.

### Immunofluorescence

The immunofluorescence assay was carried out as described earlier^32^. Briefly, MCF-7 SP and NSP cells were plated in chambered slides. MCF-7 SP cell was treated with hrCCN5 (250 ng/ml) and NSP population was treated with anti-CCN5 antibody (500 ng/ml) for 72 h and fixed in 4% paraformaldehyde for 10 min and permeabilized with 1X Triton X. After using a blocking solution, the cells were incubated with pER-α (cell signaling Inc.) for 1 h, followed by incubation with FITC conjugated secondary antibody and Alexa Fluor 555 conjugated α-tubulin at room temperature for 1 h. Cells were washed with 1X PBS and nuclei were stained with DAPI solution and mounted. Immunofluorescent-stained cells were visualized using a Nikon Eclipse TE-300 microscope.

### Clonogenic Assay

*In vitro* anchorage-dependent and independent clonogenic/colony formation assays were performed as described previously^51, 53^. Briefly, cells were treated with hrCCN5 (250 ng/ml) for 7days. Cells were then trypsinized to make single cell suspension and were re-plated in low density (10, 000 cell/well) in six-well plates to assay anchorage-dependent growth ability in the presence or absence of hrCCN5. The hrCCN5 or vehicle-treatment was continued for 10 days or until the control sets had formed sufficiently large colonies. Cells were stained with crystal violet and the plating efficiency (PE) and surviving fraction (SF) was determined as described earlier^54^.

Anchorage independent growth was determined by fluorescence-based soft agar colony forming assay using Cyto-Select 96-well Transformation assay kits (Cell BioLabs. Inc, San Diego, CA, USA). Briefly, an agar (1.2%) suspension containing BC cells were seeded over an agar (2%) underlay in 96 well plates. The treated sets (n = 5) were continued with hrCCN5 treatment for 9 days. The colonies were counted and photographed. Then agar was lysed and mixed with CyQuant and the fluorescence was read at 485/520 nm.

### Morphologic Study

Alteration of cellular morphology in hrCCN5-treated MDA-MB-231 cells was determined by the staining of actin cytoskeleton, using actin-specific dye Phalloidin-FITC. DAPI was used to stain nuclei.

### *In vitro* migration assay

Boyden chamber assay was carried out as previously (Maity *et al*.^51^). Briefly MDA-MB -231 and HCC-70 cells were treated with hrCCN5 (250 ng/ml) for 6 days and corresponding control cells was growing with the treatment set. Untreated and hrCCN5 treated cells were added on the upper Boyden chamber containing DMEM with or without hrCCN5 (250 ng/ml), and were allowed to migrate against 10% FBS in the lower chamber. The migrated cells were stained with crystal violet and solubilized with 10% acetic acid and quantified at 600 nm Specta Max 340 microplate reader (Molecular Device, Sunnyvale, CA), and calculated by SOFTmaxPRO software.

### Mammosphere Assay

Single cell suspensions of MDA-MB-231 or HCC-70 cells were prepared and seeded (0.5 cells/ml) in Mammocult media supplemented with 4 μg/ml heparin, and 0.48 μg/ml hydrocortison (Stem cell Technologies), in ultra-low attachment plates (Corning, MA, USA). Single cell suspension cultures were allowed to grow for 10 days in the presence and absence of hrCCN5 (250 ng/ml) to form mammospheres. The mammospheres were counted 7 days after plating and photographs were taken by Leica photomicroscope. The size of the spheres was measured by using NIS Element software.

### Animal Studies

To assess *in vivo* tumorigenic growth of SP, SP^(CCN5T)^ and NSP cells, sorted cells were bilaterally inoculated into subcutaneous of nude (*nu*/*nu*) mice (N = 5) in a volume of 200 μl medium/matrigel (1:1) with 0.5 × 10^5^ cells/ml (day 0). Tumor growth and size was monitored every alternative day until 20 days after inoculation using digital caliper and Studylog^R^ software (Studylog Systems, Inc. CA, USA). Nu/nu athymic mice were obtained from the Jackson Laboratories. The studies were conducted in compliance with the Institutional Animal Care and Use Committee guidelines and approved protocols of Kansas City VA Medical Center.

### Statistics

Data are represented as mean ± SD. Statistical analysis was performed as described in each corresponding Figure legend. Sample sizes are described in each corresponding Figure legend. P < 0.05 is considered significant.

## Electronic supplementary material

Supplementary Information
