# Supplementary material for: Deficiency of CCN5/WISP-2-Driven Program in breast cancer Promotes Cancer Epithelial cells to mesenchymal stem cells and Breast Cancer growth
Source: Sci Rep. 2017 Apr 27;7:1220. doi: 10.1038/s41598-017-00916-z (PMC5430628; doi:10.1038/s41598-017-00916-z)
Supplement: Supplementary file 1 — Supplementary Information [file 41598_2017_916_MOESM1_ESM.doc]

# SUPPLEMENTAL INFORMATION

**Deficiency of CCN5/WISP-2-Driven Program in breast cancer Promotes Cancer Epithelial cells to mesenchymal stem cells and Breast Cancer growth**

Amlan Das1,2#,∅, Kakali Dhar1,3≠,∅, Gargi Maity1,2∅, Sandipto Sarkar1, 4, Arnab Ghosh1,3, , Inamul Haque1,3, Gopal Dhar1,3≠,∅, Snigdha Banerjee1,3* and Sushanta K. Banerjee1,2,3,4*

INVENTORY OF SUPPLEMENTAL INFORMATION

1. **Supplemental Data**

**Contain Supplemental Figures S1 to S5 with legends**

- **Figure S1** (related to all Figures)
- **Figure S2** (related to Figure 1)
- **Figure S3** (related to Figure 4)
- **Figure S4** (related to Figure 4)
- **Figure S5** (related to Figure 6)

**
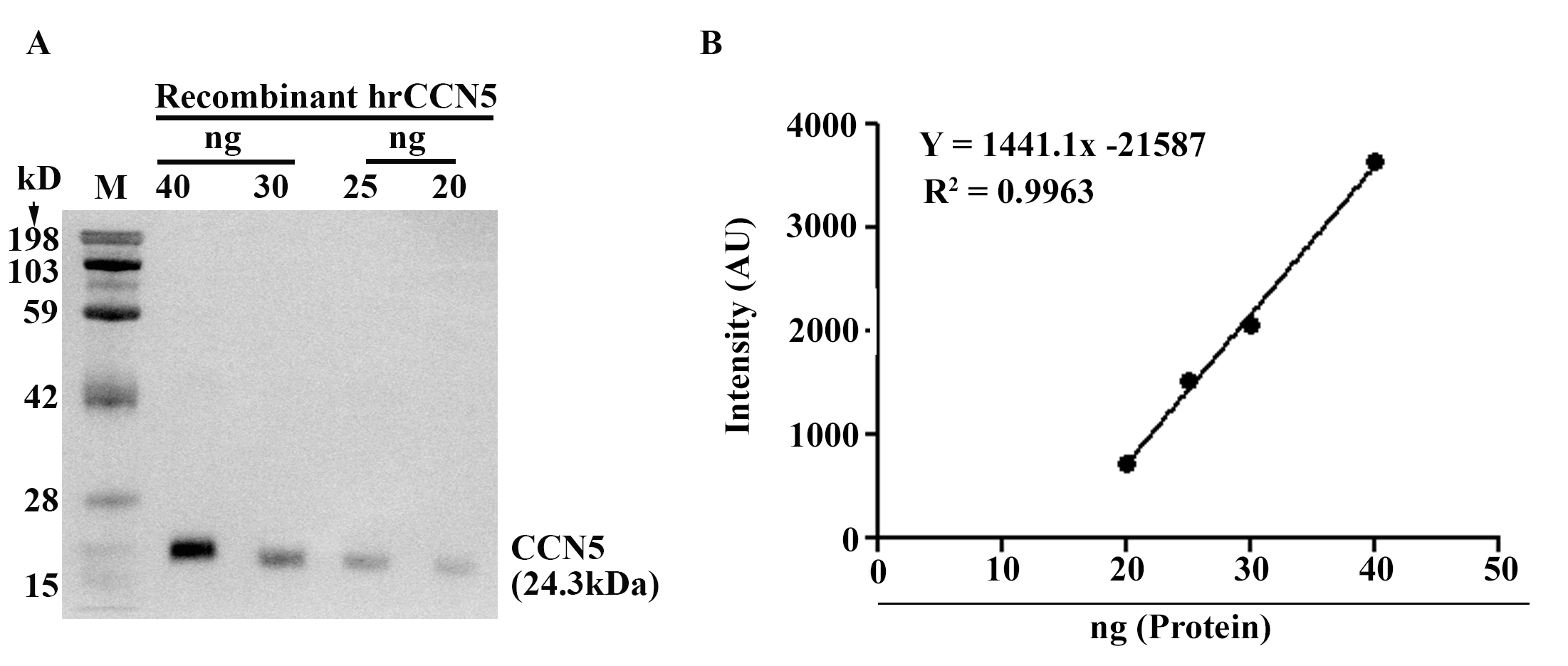
**

**Figure S1** (related to all Figures): validation of human recombinant CCN5 (hrCCN5) protein obtained commercially using Western blot analysis. **(A).** Illustrates a representative Western blot of hrCCN5 protein with different concentrations. The hrCCN5 protein was purchased from a vendor, and **(B).** Illustrates a Dose-dependent curve of hrCCN5 calculated from Western blots. The band intensities were calculated with ImageJ software. Standard curve was obtained by plotting intensity values (AU) to amount of protein load.

**Figure S2** (related to Figure 1)**: Determined the cell viability in hrCCN5-treated BC cells.**

Methods

*Cell viability Assay:* Cell viability/proliferation was measured by counting the cell numbers in HrCCN5-treated or vehicle-treated samples using Crystal Violet assay. Briefly, 50,000 cells were seeded per well in 6-well plates (N=8 for each group). Cells were treated with different doses of hrCCN5 as indicated in the Figure for 72h and stained with Crystal violet for 10 min followed by the measurement of viable cells at A540 nm on Specta Max 340 microplate reader (Molecular Device, Sunnyvale, CA), and calculated by SOFTmaxPRO software. Data represent % mean ± SD (n=8). The significant changes within variants were measured using Student t-test.

**
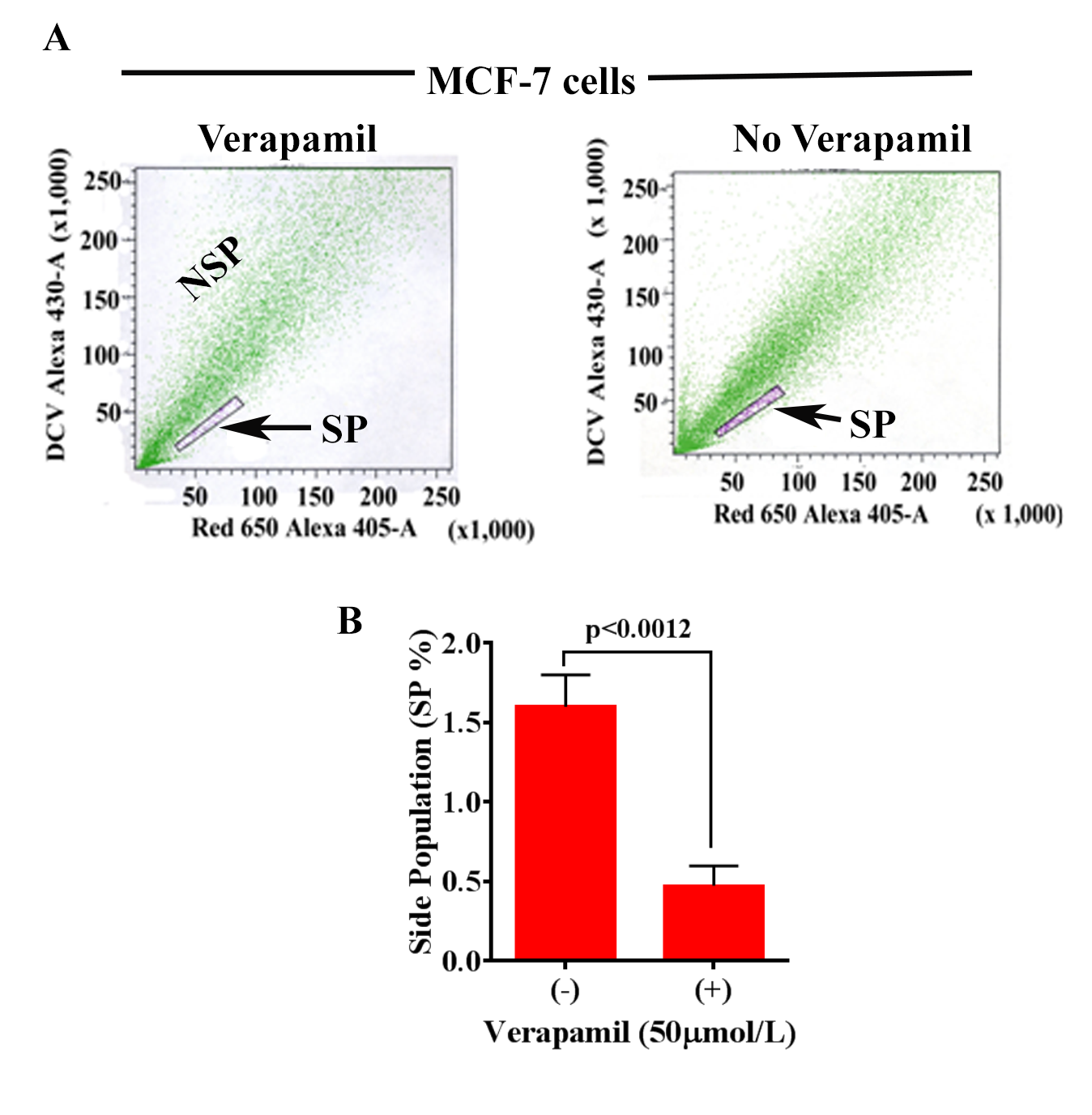
**

**Figure S3 (related to Figure 4): Isolation of SP fraction in MCF-7 breast cancer cells. Methods**

SP analysis was based on the previously described method1 with slight modifications. Briefly, cells were incubated (106 cells/ml) in pre-warmed DMEM/10% FBS cells were incubated in the presence of verapamil. After incubation, cells were spun down and re-suspended in ice-cold PBS. Propidium iodine was added to discriminate dead cells. Analysis and sorting were performed on FACS Aria IIu fluorescence-activated cell sorter (Becton Dickinson) by using a dual wavelength analysis (405 nm excitation and 440 nm emission). Data represent mean ± SD n=3). The significant changes within variants were measured using Student t-test.


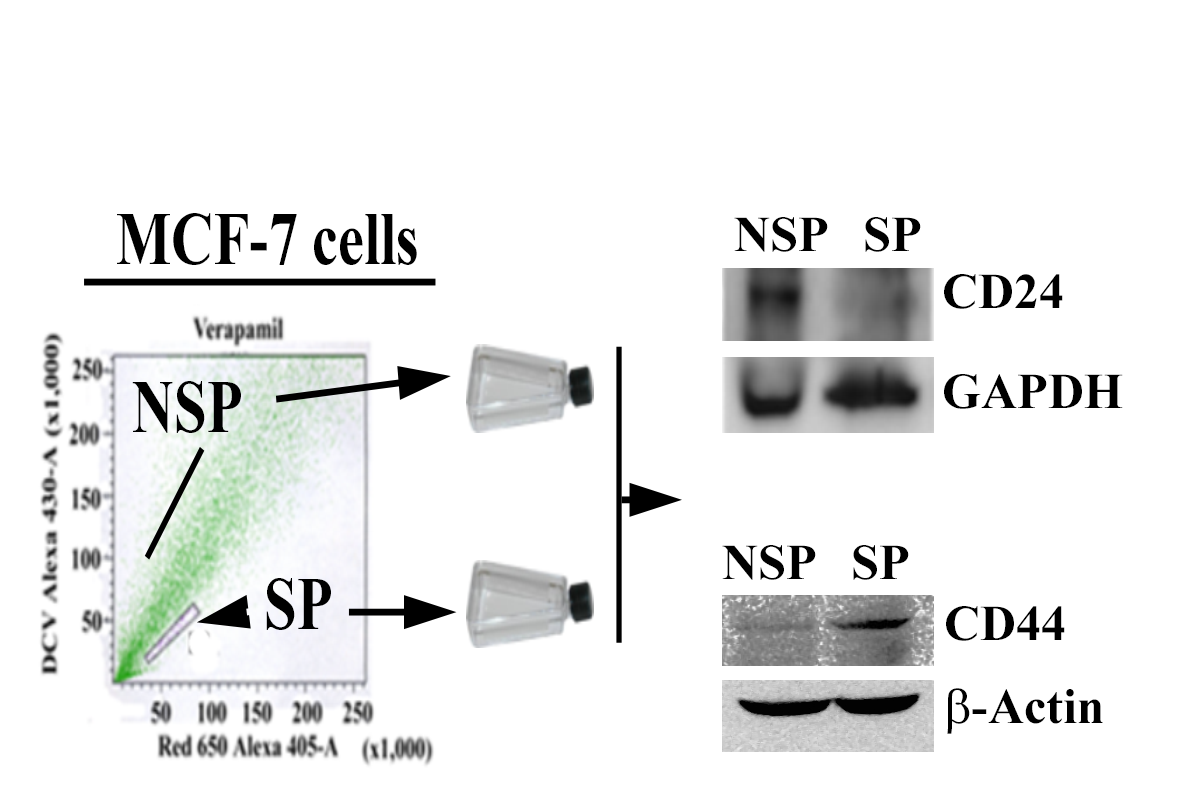


**Figure S4** (related to Figure 4): **Detection of the status of CD 24 and CD44 in SP and NSP cells using Western blot analysis**. The status was verified in three independently isolated populations by three independent investigators.

**
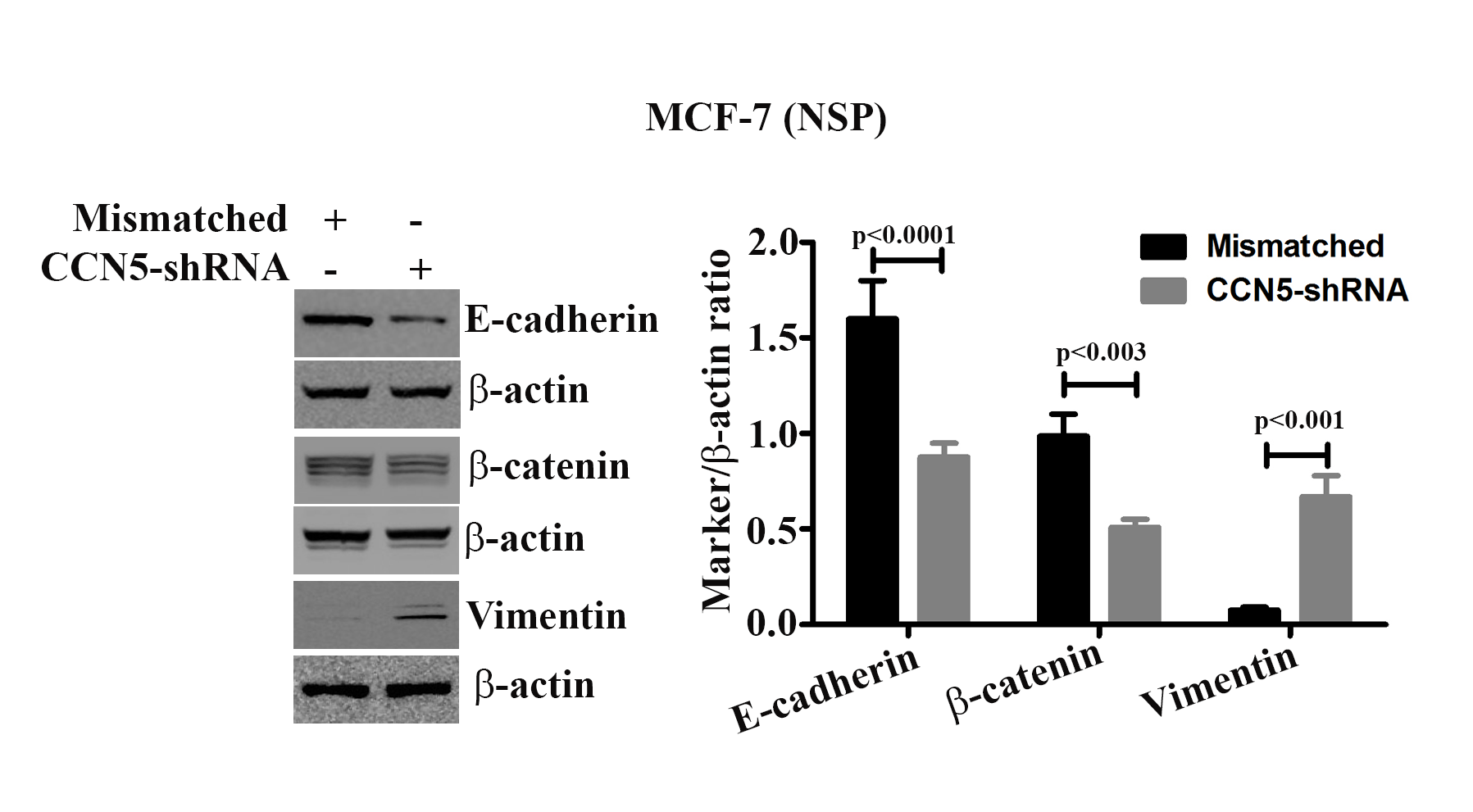
**

**Figure S5** (related to Figure 6): Detection of the status of EMT markers in CCN5-depleted NSP cells using Western blot analysis. N=3. Data represent mean ± SD. The significant changes within variants were measured using Student *t*-test.

References

1 Haque, I. et al. Cyr61/CCN1 signaling is critical for epithelial-mesenchymal transition and stemness and promotes pancreatic carcinogenesis. Molecular cancer 10, 8, doi:10.1186/1476-4598-10-8 (2011).
